# Patterns and determinants of formal healthcare utilisation among individuals with and without proxy-identified mental health need in Tanzania: A cross-sectional analysis of the National Panel Survey

**DOI:** 10.1371/journal.pmen.0000644

**Published:** 2026-09-17

**Authors:** Gallen Mlenge, Odass Bilame, Lutengano Mwinuka

**Affiliations:** 1 Department of Business Management, Mbeya University of Science and Technology, Mbeya, Tanzania; 2 Department of Economics, University of Dodoma, Dodoma, Tanzania; Northern Ontario School of Medicine: NOSM University, CANADA

## Abstract

Formal healthcare utilisation remains persistently low across sub-Saharan Africa, yet utilisation patterns among individuals with mental health needs are poorly understood in low- and middle-income country contexts. Using data from the fifth wave of the Tanzania National Panel Survey (2020/21), this study examined patterns and determinants of formal healthcare utilisation among individuals aged 15 years and above with and without proxy-identified mental health need. A nationally representative cross-sectional sample of 11,891 individuals with non-missing outcome data was analysed. Proxy-identified mental health need was determined using the Washington Group Short Set item on difficulty remembering or concentrating at the severe threshold, a proxy associated with common mental disorders. The outcome was any formal provider visit in the four weeks prior to the interview, regardless of reason. Survey-weighted logistic regression estimated odds ratios and predicted probabilities within the Andersen Behavioural Model. Missing outcome data affected 505 individuals in a Missing Not At Random pattern; extreme bounds sensitivity analysis assessed implications for subgroup estimates. Overall weighted formal healthcare utilisation was 19.2%. Proxy-identified mental health need was observed in 54 individuals (weighted prevalence 0.45%). Female sex, older age (55 and above), and Southern Highlands zone residence were independently associated with higher utilisation. Household wealth showed a strong dose-response gradient: individuals in the richest quintile had 2.71 times higher adjusted odds than those in the poorest. Proxy-identified mental health need was not significantly associated with utilisation (adjusted odds ratio 1.27; 95% confidence interval: 0.53–3.04; p = 0.588). Extreme bounds analysis indicated that true utilisation could range from 2.0% to 92.7%, confirming subgroup findings are exploratory. Formal healthcare utilisation in Tanzania is strongly patterned by household wealth, sex, age, and geographic zone. Addressing wealth-related barriers and strengthening service availability in lower-utilisation zones should be the priorities for advancing equitable healthcare utilisation in Tanzania.

## Introduction

Mental health disorders are among the leading causes of disability globally, accounting for a substantial share of years lived with disability across all age groups [1]. Despite growing recognition of their public health importance, access to and utilisation of formal health services remains limited in many low- and middle-income countries, with large treatment gaps particularly pronounced in sub-Saharan Africa [2]. Shortages of trained providers, widespread stigma, weak health system infrastructure, geographic barriers, and financial constraints collectively constrain formal healthcare utilisation in this region [2–4].

Tanzania faces a dual burden of communicable and non-communicable diseases, with mental health conditions increasingly recognised as a major contributor to population morbidity [5]. National policies acknowledge mental health as a priority area, yet service availability remains concentrated in urban centres and specialised tertiary facilities, and community-based mental health infrastructure remains limited in scope and reach [6]. With fewer than 0.1 psychiatrists per 100,000 population and no community-based mental health system of any meaningful scale, most individuals with mental health conditions have no realistic prospect of receiving specialist care [7]. Understanding who accesses formal healthcare and who does not is therefore essential for informing health system strengthening and equitable policy development.

The determinants of formal health care utilisation in Tanzania are complex and context-specific. The Andersen Behavioural Model of Health Services Use conceptualises utilisation as a function of predisposing factors such as age, sex, and education; enabling factors such as household wealth, geographic access, and employment; and need factors including health status and perceived morbidity [8]. Income, sex, age, urban residence, and distance to facilities are consistently identified as determinants of general healthcare utilisation across sub-Saharan Africa [9]. For individuals with mental health needs, additional barriers intensify these effects: widespread stigma, explanatory models that attribute psychological distress to supernatural causes, and preference for traditional and faith-based healers over formal medical practitioners further reduce the likelihood of formal provider contact [10,11]. Although theory positions need as the primary driver of healthcare utilisation, evidence from low- and middle-income countries consistently shows that enabling factors, particularly household wealth and geographic access, are stronger predictors of actual utilisation, generating systematic treatment gaps for the most vulnerable populations [3,12].

Most studies of healthcare utilisation in Tanzania have focused on infectious diseases or maternal health. The few that have examined mental health have typically been small, facility-based, or restricted to adults aged 18 and above, limiting generalisability [10,13]. Nationally representative evidence on formal healthcare utilisation among individuals with mental health need is scarce, and few studies have specifically examined whether socioeconomic inequalities in utilisation differ between individuals with and without such need [13]. There is increasing recognition that individuals aged 15–17 years carry a measurable burden of mental health morbidity, and excluding them from population-based analyses underestimates the scale of the problem [14–17]. This study includes individuals aged 15 years and above and uses a population-level proxy indicator for mental health need to partially address this gap.

Taken together, the evidence points to a substantial and inequitably distributed burden of mental health need in Tanzania, concentrated among populations already facing the greatest structural barriers to formal healthcare access, yet studied primarily through small and non-representative samples. Against this background, this study draws on nationally representative data from the Tanzania National Panel Survey to address three specific objectives: to describe the prevalence and sociodemographic patterning of formal healthcare utilisation in the full analytic sample; to identify independent determinants of formal healthcare utilisation using multivariable logistic regression within the Andersen Behavioural Model framework; and to describe the profile and formal healthcare utilisation of the subgroup with proxy-identified mental health need, with explicit acknowledgement of the data limitations affecting subgroup inference.

## Materials and methods

### Study design and data source

This is a secondary cross-sectional analysis using data from the fifth wave of the Tanzania National Panel Survey (NPS Wave 5, 2020/21), a nationally representative household survey conducted between 2020 and 2021 by the National Bureau of Statistics (NBS) of Tanzania in partnership with the World Bank Living Standards Measurement Study programme [18,19]. The NPS Wave 5 employed a stratified two-stage cluster sampling design, with households selected through probability proportional to size sampling from enumeration areas defined in the 2012 national census. The sampling frame covered 51 strata across mainland Tanzania and Zanzibar, with 417 primary sampling units in the main analytic sample. All usual household members were eligible for interview.

### Study population

All individuals aged 15 years and above at the time of interview were eligible for inclusion. The age threshold of 15 years was chosen to capture adolescents capable of participating in or influencing healthcare decisions, and who carry a measurable burden of mental health morbidity that population analyses restricted to adults aged 18 and above would systematically underestimate [14–16].

Two groups were excluded from the analytic sample. First, the NPS Wave 5 included an urban booster sample, an additional set of households deliberately oversampled in urban areas of five mainland regions (Dodoma, Arusha, Tanga, Mbeya, and Mwanza) to improve the precision of urban estimates which used a different sampling frame from the main panel survey. These booster households lacked both primary sampling unit and stratum identifiers required for design-based variance estimation and could not be incorporated into the survey design; they were therefore excluded from all analyses (n = 1,296 individuals aged 15 and above). Second, a further 505 individuals (4.1%) had missing values on the outcome variable in a pattern consistent with Missing Not At Random, as detailed in the missing data subsection below, and were excluded from the regression analytic sample.

Following these exclusions, the main analytic sample after booster removal comprised 12,396 individuals aged 15 years and above with complete survey design information, representing a weighted population of 32,400,437. The final regression analytic sample after further exclusion of those with missing outcome data was 11,891 individuals, corresponding to a weighted population of 31,050,573.

### Outcome variable

The primary outcome was formal healthcare utilisation, defined as visiting any formal healthcare provider in the four weeks preceding the survey (yes = 1; no = 0), derived from the NPS Wave 5 question: “Has [NAME] visited a healthcare provider in the last 4 weeks?” This is a self-reported measure, with responses provided by the individual or, where the individual was unavailable, by a knowledgeable household member acting as a proxy respondent, as is standard practice in nationally representative household surveys in low- and middle-income countries [18].

### Exposure variable

The primary variable of interest was proxy-identified mental health need, constructed from the Washington Group Short Set question: “Does [NAME] have difficulty remembering or concentrating?” Responses were dichotomised into no proxy-identified mental health need (codes 1–3: no difficulty at all, no difficulty with assistive device, or some difficulty) and proxy-identified mental health need (codes 4–5: a lot of difficulty, or cannot perform at all). This threshold is consistent with the Washington Group recommended approach for identifying functional difficulty in population surveys, which uses the severe categories of “a lot of difficulty” and “cannot do at all” to define the positive group [20]. Individuals reporting “some difficulty” were classified as not having proxy-identified mental health need, ensuring the positive group captures only substantial functional impairment rather than mild or moderate cognitive symptoms, consistent with prior use of this item in low- and middle-income country household surveys [15,16].

The use of this item as a proxy indicator of mental health need is supported by a convergent body of evidence across population surveys. Cognitive symptoms specifically difficulty remembering and concentrating are among the most consistently reported functional manifestations of common mental disorders, particularly depression and anxiety. In a prospective cohort study of 623 adults with recurrent major depressive disorder, persistent cognitive difficulty was strongly and independently associated with depression severity and functional impairment across multiple assessment points, with impaired attention and working memory being particularly linked to poor daily functioning [21]. Difficulty concentrating is a recognised diagnostic criterion for generalised anxiety disorder and has been shown to have independent incremental utility in identifying anxiety-related functional impairment beyond worry alone [22]. At the population level, adults with cognitive disability identified using the same functional difficulty question used in this study show the highest prevalence of frequent mental distress of any disability type in nationally representative surveys, with over half of those with combined cognitive and mobility disability reporting frequent mental distress [23]. In population-based surveys across five low- and middle-income countries, adults with physical and sensory functional difficulties assessed using Washington Group items showed substantially elevated odds of anxiety and depression, with adjusted odds ratios ranging from 2.0 to 14.2, suggesting that Washington Group functional difficulty items more broadly capture populations at elevated mental health risk [24].

At the same time, this item is not a validated mental health screening instrument. The cognitive difficulty question may also capture neurodevelopmental conditions, neurological impairment, dementia, and other causes of cognitive difficulty distinct from psychiatric disorders. In population surveys, the item has been shown to miss a substantial proportion of individuals with serious mental illness, partly because many people with mental health conditions do not report severe functional difficulty in the cognitive domain [25]. The Washington Group on Disability Statistics acknowledges that the Short Set may identify some persons with psychosocial disabilities, those whose difficulties manifest as problems with cognition, communication, and self-care, but it does not include all persons with psychosocial disabilities and does not contain a dedicated psychosocial functioning domain [26]. Proxy-identified mental health need in this study should therefore be understood as identifying a population with severe cognitive functional difficulty that is epidemiologically associated with but not equivalent to clinical mental health conditions. It should not be interpreted as equivalent to a clinical diagnosis, and findings are described throughout as pertaining to individuals with proxy-identified mental health need rather than to individuals with clinically confirmed mental disorders.

In descriptive terms, this study examines formal healthcare utilisation among individuals with and without severe difficulty remembering or concentrating as measured by a single Washington Group Short Set item. The label “proxy-identified mental health need” is used as shorthand throughout, reflecting the item’s established epidemiological associations with common mental disorders while acknowledging that the underlying construct is broader than any specific psychiatric condition.

### Covariates

Covariates were specified a priori within the three domains of the Andersen Behavioural Model of Health Services Use [8]: predisposing factors, enabling factors, and the need factor.

Predisposing factors included sex (male; female), age group (15–24 years; 25–34; 35–44; 45–54; 55 and above), highest education level attained (no education; primary incomplete; primary complete; secondary and above), and marital status (never married; married or cohabiting; divorced or separated; widowed). Education was constructed from the NPS Wave 5 grade completion variable using the Tanzanian education system classification: primary incomplete corresponded to Standard 1–6; primary complete to Standard 7; and secondary and above to Form 1 onwards, including diploma and university levels. For individuals with missing grade information who had attended school, a fall-back classification using the NPS literacy variable was applied, assigning primary incomplete to those with any literacy and no education to those reporting inability to read or write.

Enabling factors included household wealth quintile, place of residence, geographic zone, and employment sector. Household wealth was proxied by real total consumption expenditure per adult equivalent standardised over 28 days, categorised into quintiles (poorest Q1 through richest Q5) constructed using survey-weighted estimation. Place of residence was dichotomised as rural or urban. Geographic zone was constructed from NPS region codes following the National Bureau of Statistics classification into seven mainland zones (Lake, Western, Northern, Central, Eastern, Southern Highlands, and Southern) and Zanzibar, consistent with standard NBS reporting. Employment sector was categorised into five groups: formal wage employment (government and parastatal employers); informal wage employment (private sector and other employers); non-farm self-employment; agricultural (own farm or unpaid family agricultural work); and not working or other. This classification was constructed using both the last seven days and last 12 months employment modules, with priority given to last seven days activity and 12-month recall used as a supplement for those with no recorded last seven days activity.

Three variables were considered but excluded from the model with explicit justification. Health insurance was not available for the full analytic sample in the NPS Wave 5 public release, being captured only conditionally for wage employees through the employment module and for care-seekers through the health module, precluding its use as a universal covariate. Distance to the nearest health facility could not be constructed because sub-district geographic identifiers (ward, village, and enumeration area codes) were withheld from the public release by NBS for respondent confidentiality; urban or rural residence and geographic zone dummies serve as geographic access proxies. A general recent illness variable was not available for all adults aged 15 and above in the NPS Wave 5; proxy-identified mental health need serves as the need indicator in the model.

### Missing data

The outcome variable had 505 missing values (4.1% of the main analytic sample of 12,396). Examination of the missing data pattern revealed that all 505 missing values on the outcome were also missing on the proxy-identified mental health need variable, with zero missing outcome values among the 11,837 individuals with non-missing proxy-identified mental health need. This perfect concentration of missing outcome data within the group with missing proxy-identified mental health need is consistent with Missing Not At Random (MNAR), whereby the probability of missing outcome data is related to the unobserved value itself [27,28]. Possible explanations include data collection difficulties associated with cognitive impairment, fieldwork inconsistencies, or questionnaire routing errors in the Computer-Assisted Personal Interviewing (CAPI) instrument.

To assess the robustness of subgroup estimates under different assumptions about the missing data, extreme bounds analysis was conducted following the approach of Manski (1990) [29]. Three scenarios were constructed: the observed estimate using only the 54 individuals with proxy-identified mental health need and non-missing outcome (S0); a lower bound assuming all 505 individuals with missing data had not visited a formal provider (S1); and an upper bound assuming all 505 had visited a formal provider (S2). Pattern-of-missingness analysis was also conducted, comparing the 505 individuals with missing outcome against the 11,891 with non-missing outcome on all observable sociodemographic characteristics using t-tests and Pearson chi-squared tests.

### Data analysis

All analyses were conducted in Stata version 17.0 (StataCorp LLC, College Station, TX, USA). The complex survey design was declared using the svyset command, specifying household sampling weights (hhweight), primary sampling unit clustering (cluster), and stratification (strata), with linearised Taylor variance estimation and the singleunit (centered) option to handle strata containing a single primary sampling unit. This approach ensures that all point estimates, standard errors, and confidence intervals appropriately reflect the probability sampling design of the NPS Wave 5.

The analysis proceeded in three parts. Part one produced survey-weighted descriptive statistics describing the distribution of formal healthcare utilisation and all covariates across the full analytic sample (n = 11,891). Bivariate associations between each covariate and formal healthcare utilisation were assessed using the Rao-Scott design-based F-test, which is appropriate for complex survey data.

Part two fitted survey-weighted logistic regression models to identify independent determinants of formal healthcare utilisation. Unadjusted (crude) odds ratios were estimated for each predictor individually. A single adjusted model was then fitted including all covariates simultaneously, as shown in equation 1:


$$\begin{array}{c} \begin{array}{c} \text{log}\left(\frac{\rho_{i}}{\text{1}-\rho_{i}}\right)=\beta_{\text{0}}+\beta_{\text{1}}(MH\_proxy)+\beta_{\text{2}}(Sex)+\\ \beta_{\text{3}}(Age\_group)+\beta_{\text{4}}(Education)+\\ \beta_{\text{5}}(Marital\_status)+\beta_{\text{6}}(wealth\_qu\text{int}ile)+\\ \beta_{\text{7}}(\text{Re}sidence)+\beta_{\text{8}}(Zone)+\\ \beta_{\text{9}}(Employment\_\text{sec}tor)+\varepsilon \end{array} \end{array}$$ (1)

Where ρ is the probability that individual i visited a formal healthcare provider in the preceding four weeks prior to the interview; β_0_ is the intercept; β_1_ through β_9_ are the regression coefficients for each predictor; and ε is the error term. The model was specified a priori on theoretical grounds using the Andersen Behavioural Model, with all nine predictors included simultaneously regardless of their bivariate significance. Reference categories were: male (sex); 15–24 years (age group); no education (education); never married (marital status); poorest quintile Q1 (wealth quintile); rural (residence); Lake zone (zone); and not working or other (employment sector).

To aid interpretation, survey-weighted adjusted predicted probabilities were estimated for each predictor using the Stata margins command following the adjusted model, as shown in equation 2.


$$\begin{array}{c} \hat{P}\left(Y=\text{1}|X_{k}=x\right)=\frac{\text{1}}{n}\sum_{i=\text{1}}^{n}\frac{\text{exp}\left({\hat{\beta }}_{\text{0}}+{\hat{\beta }}_{k}x+\sum_{j\neq k}{\hat{\beta }}_{j}X_{ij}\right)}{\text{1}+\text{exp}\left({\hat{\beta }}_{\text{0}}+{\hat{\beta }}_{k}x+\sum_{j\neq k}{\hat{\beta }}_{j}X_{ij}\right)} \end{array}$$ (2)

Where $\hat{P}\left(Y=1|X_{k}=x\right)$ is the estimated probability of formal healthcare utilisation when predictor $X_{k}$ takes value x; Y=1 indicates that the individual visited a formal healthcare provider; $X_{k}$ is the predictor of interest evaluated at value x; n is the total number of individuals in the analytic sample (11,891); i is the index for each individual; ${\hat{\beta }}_{\text{0}}$ is the estimated intercept from the adjusted logistic regression model; ${\hat{\beta }}_{k}$ is the estimated coefficient for the predictor of interest; ${\hat{\beta }}_{j}$ are the estimated coefficients for all other covariates j≠k; $X_{ij}$ is the observed value of covariate j for individual i, held at their actual values; exp(.) is the exponential function; and $\frac{\text{1}}{n}$ denotes averaging over all individuals in the sample, producing a marginal population-averaged predicted probability. This approach, implemented using the Stata margins command following the adjusted survey-weighted logistic regression model, produces predicted probabilities averaged over the observed covariate distribution of the full analytic sample and is reported for all modelled predictors including place of residence, regardless of statistical significance in the adjusted model.

Model fit was assessed using the Hosmer-Lemeshow goodness-of-fit test applied to the unweighted logistic regression model, and multicollinearity was assessed using variance inflation factors. Statistical significance was defined as p < 0.05 throughout.

Part three produced a weighted descriptive profile of the subgroup with proxy-identified mental health need (n = 54 with non-missing outcome), including formal healthcare utilisation by sociodemographic characteristic. Multivariable regression was not pursued in this subgroup because only 54 individuals with proxy-identified mental health need had non-missing outcome data, which is insufficient for stable adjusted estimation. All subgroup findings are explicitly framed as exploratory and are reported alongside their confidence intervals to convey the high degree of uncertainty. The extreme bounds sensitivity analysis results are reported in a S1 Table. This study is reported in accordance with the Strengthening the Reporting of Observational Studies in Epidemiology (STROBE) guidelines as shown in S2 Table [30].

### Ethical consideration

This study involved secondary analysis of a publicly available, de-identified dataset held in the Tanzania National Bureau of Statistics microdata repository (https://microdata.nbs.go.tz/index.php/catalog/35/get-microdata). No individual participants can be identified from the dataset, and the authors had no access to information that could identify individual participants during or after data collection. The dataset was first accessed for research purposes in 11 August 2025. Formal written permission to use the dataset for this study was subsequently obtained from NBS in March 2026, and institutional ethics clearance was granted by the University of Dodoma Institutional Research Review Ethics Committee (IRREC) in March 2026.

## Results

### Sociodemographic characteristics and formal healthcare utilisation in the full analytic sample

The main analytic sample comprised 12,396 individuals aged 15–95 years after excluding the urban booster sample (n = 1,296) which lacked the design identifiers required for survey-weighted estimation. A further 505 individuals (4.1%) had missing values on the outcome variable in a pattern consistent with Missing Not At Random, as detailed in the missing data subsection of the Methods section. The final regression analytic sample was therefore 11,891 individuals representing a weighted population of approximately 31 million Tanzanians. Table 1 presents the weighted sociodemographic distribution and formal healthcare utilisation prevalence of this sample.

**Table 1:** Sociodemographic characteristics and weighted prevalence of formal healthcare utilisation in the full analytic sample (N = 11,891), Tanzania NPS 2020/2021.

Characteristic	n (weighted %)	Utilisation % (95% CI)	p-value^†^
**Total sample**	11,891	19.2 (18.1–20.3)	–
**Mean age, years (95% CI)**	34.3 (33.9–34.6)	–	–
**Age group (years)**			**<0.001**
15–24	4,158 (33.6%)	14.0 (12.6–15.5)	
25–34	2,703 (24.3%)	20.5 (18.5–22.8)	
35–44	1,978 (18.1%)	20.1 (17.7–22.6)	
45–54	1,401 (11.9%)	20.0 (17.4–22.9)	
≥ 55	1,651 (12.0%)	28.9 (26.0–31.9)	
**Sex**			**<0.001**
Male	5,692 (47.8%)	15.3 (14.0–16.6)	
Female	6,199 (52.2%)	22.8 (21.3–24.3)	
**Place of residence**			**<0.001**
Rural	7,397 (72.0%)	17.4 (16.2–18.7)	
Urban	4,494 (28.0%)	23.7 (21.7–25.8)	
**Wealth quintile**			**<0.001**
Poorest (Q1)	2,204 (19.8%)	10.8 (9.2–12.7)	
Poorer (Q2)	2,322 (20.3%)	15.5 (13.5–17.7)	
Middle (Q3)	2,459 (19.9%)	19.5 (17.3–21.8)	
Richer (Q4)	2,598 (20.0%)	22.8 (20.4–25.5)	
Richest (Q5)	2,308 (20.1%)	27.3 (24.9–29.9)	
**Education level**			**<0.001**
No education	1,965 (16.5%)	17.4 (14.9–20.1)	
Primary incomplete	3,328 (27.0%)	16.7 (15.1–18.5)	
Primary complete	3,804 (38.5%)	20.0 (18.2–21.9)	
Secondary and above	2,794 (18.1%)	22.9 (20.7–25.3)	
**Marital status**			**<0.001**
Never married	3,858 (31.3%)	12.8 (11.3–14.3)	
Married/cohabiting	6,382 (55.5%)	21.3 (19.8–22.8)	
Divorced/separated	900 (7.6%)	22.9 (19.5–26.7)	
Widowed	751 (5.7%)	29.3 (25.1–34.0)	
**Employment sector**			**<0.001**
Formal wage	270 (2.0%)	23.5 (15.8–33.5)	
Informal wage	1,980 (17.2%)	18.2 (16.0–20.7)	
Self-employed non-farm	1,846 (16.8%)	26.8 (24.2–29.5)	
Agricultural	5,143 (48.3%)	16.9 (15.5–18.4)	
Not working/other	2,652 (15.8%)	18.7 (16.5–21.0)	
**Geographic zone**			**<0.001**
Lake	2,924 (26.9%)	15.2 (13.4–17.2)	
Western	1,133 (9.8%)	15.1 (12.0–18.8)	
Northern	1,080 (13.7%)	14.7 (12.0–17.8)	
Central	503 (6.4%)	15.2 (11.0–20.6)	
Eastern	2,036 (19.2%)	22.7 (20.2–25.4)	
Southern Highlands	1,332 (15.8%)	30.0 (26.5–33.9)	
Southern	765 (5.2%)	18.4 (15.9–21.3)	
Zanzibar	2,118 (3.1%)	19.5 (16.6–22.8)	
**Proxy-identified mental health need ‡**			**0.693**
No Proxy-identified MH need	11,837 (99.6%)	19.2 (18.1–20.3)	
Proxy-identified MH need	54 (0.4%)	22.0 (10.8–39.7)	

**Abbreviations:** CI, confidence interval; MH, mental health; NPS, National Panel Survey.

† p-values from Rao-Scott design-based F-test; the p-value is reported for the first category and applies to the overall test for each variable. ‡ Proxy-identified mental health need is derived from the Washington Group Short Set item on difficulty remembering or concentrating (severe threshold: a lot of difficulty or cannot perform at all). The n = 54 observed cases are from the 11,891 with non-missing outcome. A further 505 individuals had missing data on both the outcome and the proxy item (Missing Not At Random); the weighted prevalence of 0.45% (95% CI: 0.32–0.61%) is estimated from those with non-missing data and likely underestimates the true population prevalence. Formal healthcare utilisation = any formal provider visit in the preceding four weeks, regardless of reason.

The mean weighted age was 34.3 years (95% CI: 33.9–34.6). The 15–24 age group was the largest, accounting for 33.6% of the weighted sample, with the 25–34 group contributing a further 24.3%. Just over half of participants were female (52.2%). The sample was predominantly rural, with 72.0% residing in rural areas, reflecting Tanzania’s demographic structure. Household wealth was approximately evenly distributed across quintiles, each accounting for roughly 20% of the weighted sample. The Lake zone was the most represented geographic zone (26.9%), followed by the Eastern zone including Dar es Salaam (19.2%). Agricultural employment was the most common employment category (48.3%), while formal wage employment accounted for only 2.0% of the sample.

The overall weighted prevalence of formal healthcare utilisation, any formal provider visit in the preceding four weeks regardless of reason was 19.2% (95% CI: 18.1–20.3%), meaning approximately one in five Tanzanians aged 15 and above had contact with a formal health provider during that period. Statistically significant differences in formal healthcare utilisation were observed across all sociodemographic characteristics (all p < 0.001), with the single exception of proxy-identified mental health need (p = 0.693). Female participants had substantially higher utilisation than males (22.8% versus 15.3%). Utilisation increased with age, from 14.0% in the youngest group to 28.9% among those aged 55 and above. Urban residents utilised formal healthcare more frequently than rural residents (23.7% versus 17.4%). The wealth gradient was steep and consistent: utilisation increased monotonically from 10.8% in the poorest quintile to 27.3% in the richest, a difference of more than 16 percentage points. Southern-Highlands zone had the highest utilisation rate (30.0%), while Northern zone had the lowest (14.7%). Individuals with proxy-identified mental health need did not utilise formal healthcare at a significantly different rate from those without (22.0% versus 19.2%; Rao-Scott F = 0.16, p = 0.693).

Proxy-identified mental health need was observed in 54 individuals among those with non-missing outcome data, corresponding to a weighted prevalence of 0.45% (95% CI: 0.32–0.61%) and an estimated 138,311 individuals nationally (95% CI: 93,088–183,534). Given the Missing Not At Random structure of the missing data, this figure likely underestimates the true prevalence; 505 individuals had missing data on both the outcome and the proxy-identified mental health need item and could not be characterised.

### Determinants of formal healthcare utilisation: multivariable logistic regression and adjusted predicted probabilities

Table 2 presents crude and adjusted odds ratios alongside survey-weighted adjusted predicted probabilities from the logistic regression model. The adjusted model included all nine predictors simultaneously in line with the a priori Andersen Behavioural Model specification. The model fitted the data adequately. Hosmer-Lemeshow chi-squared (8) = 11.95, p = 0.153 and no meaningful multicollinearity was identified (mean variance inflation factor = 2.45; maximum individual variance inflation factor = 6.74 for agricultural employment, reflecting expected correlations between employment, wealth, and rural residence in the Tanzanian context.

**Table 2:** Crude and adjusted odds ratios and survey-weighted adjusted predicted probabilities for determinants of formal healthcare utilisation (N = 11,891), Tanzania NPS 2020/2021.

Variable	Crude OR	95% CI	p	Adj. OR	95% CI	p	Pred. prob. (95% CI)
**Proxy-identified MH need**							
No (ref)	1.00	–	–	1.00	–	–	19.2% (18.1–20.2%)
Yes	1.19	0.51–2.78	0.693	1.27	0.53–3.04	0.588	22.9% (8.7–37.0%)
**Sex**							
Male (ref)	1.00	–	–	1.00	–	–	15.6% (14.2–16.9%)
Female	1.63	1.45–1.84	<0.001	1.61	1.40–1.85	<0.001	22.4% (20.9–23.9%)
**Age group (years)**							
15–24 (ref)	1.00	–	–	1.00	–	–	17.1% (15.0–19.1%)
25–34	1.58	1.33–1.89	<0.001	1.12	0.90–1.39	0.299	18.6% (16.6–20.7%)
35–44	1.54	1.28–1.86	<0.001	1.07	0.85–1.37	0.554	18.0% (15.8–20.3%)
45–54	1.53	1.25–1.87	<0.001	1.13	0.88–1.46	0.344	18.8% (16.1–21.4%)
≥ 55	2.49	2.05–3.01	<0.001	1.92	1.50–2.45	<0.001	27.5% (24.4–30.6%)
**Place of residence**							
Rural (ref)	1.00	–	–	1.00	–	–	19.1% (17.7–20.4%)
Urban	1.47	1.28–1.69	<0.001	1.03	0.85–1.24	0.784	19.4% (17.3–21.6%)
**Wealth quintile**							
Poorest Q1 (ref)	1.00	–	–	1.00	–	–	11.9% (10.0–13.9%)
Poorer Q2	1.51	1.20–1.90	<0.001	1.42	1.12–1.81	0.004	16.0% (13.9–18.1%)
Middle Q3	1.99	1.59–2.48	<0.001	1.79	1.42–2.25	<0.001	19.2% (17.0–21.3%)
Richer Q4	2.43	1.94–3.06	<0.001	2.12	1.66–2.70	<0.001	21.8% (19.4–24.2%)
Richest Q5	3.09	2.50–3.83	<0.001	2.71	2.13–3.46	<0.001	26.1% (23.4–28.7%)
**Geographic zone**							
Lake (ref)	1.00	–	–	1.00	–	–	16.9% (14.8–19.0%)
Western	0.99	0.73–1.34	0.945	1.06	0.79–1.42	0.719	17.6% (13.9–21.4%)
Northern	0.96	0.73–1.27	0.777	0.81	0.62–1.05	0.113	14.3% (11.8–16.8%)
Central	1.00	0.67–1.49	0.991	0.95	0.64–1.42	0.817	16.3% (11.4–21.2%)
Eastern	1.64	1.33–2.03	<0.001	1.15	0.89–1.51	0.286	18.9% (16.3–21.6%)
Southern Highlands	2.40	1.90–3.03	<0.001	2.22	1.76–2.81	<0.001	30.2% (26.7–33.7%)
Southern	1.26	1.00–1.59	0.052	1.11	0.86–1.42	0.411	18.4% (15.5–21.2%)
Zanzibar	1.36	1.06–1.73	0.015	1.04	0.78–1.40	0.767	17.5% (14.4–20.6%)
**Employment sector**							
Not working/other (ref)	1.00	–	–	1.00	–	–	19.0% (16.5–21.5%)
Formal wage	–	–	–	–	–	–	17.3% (10.0–24.5%)
Informal wage	0.73	0.43–1.22	0.224	1.12	0.65–1.96	0.678	18.9% (16.4–21.4%)
Self-employed non-farm	1.19	0.71–2.01	0.512	1.41	0.80–2.47	0.232	22.3% (20.1–24.5%)
Agricultural	0.66	0.40–1.09	0.105	1.07	0.61–1.85	0.821	18.1% (16.7–19.6%)

**Abbreviations:** OR, odds ratio; Adj. OR, adjusted odds ratio; CI, confidence interval; Pred. prob., survey-weighted adjusted predicted probability; ref, reference category; MH, mental health; VIF, variance inflation factor. Predicted probabilities are average marginal predictions from the adjusted survey-weighted logistic regression model, computed using the Stata margins command with all other covariates held at their observed values. Predicted probabilities are reported for all modelled predictors including place of residence, regardless of statistical significance in the adjusted model. Formal healthcare utilisation = any formal provider visit in the preceding four weeks, regardless of reason.

In the unadjusted analysis, proxy-identified mental health need showed no significant association with formal healthcare utilisation (crude OR: 1.19, 95% CI: 0.51–2.78, p = 0.693). All other predictors were significantly associated with formal healthcare utilisation in the expected direction in crude analyses, with the exception of employment sector which showed heterogeneous patterns across categories.

After full adjustment, five predictors remained independently and significantly associated with formal healthcare utilisation. Female sex was associated with 61% higher adjusted odds compared to male (aOR: 1.61, 95% CI: 1.40–1.85, p < 0.001), with adjusted predicted probabilities of 15.6% for men and 22.4% for women, a gap of 6.8 percentage points persisting after controlling for all other covariates. The age gradient showed a distinct pattern in the adjusted model. Compared to the youngest group (15–24 years), those aged 25–34, 35–44, and 45–54 years did not have significantly higher adjusted odds of formal healthcare utilisation (aORs ranging from 1.07 to 1.13, all p > 0.29), suggesting that the unadjusted age gradient across these middle groups is largely attributable to compositional differences in wealth, marital status, and employment rather than age itself. Only those aged 55 years and above retained a significant independent association (aOR: 1.92, 95% CI: 1.50–2.45, p < 0.001), with an adjusted predicted probability of 27.5% compared to 17.1% in the youngest group.

Education was independently associated with utilisation after adjustment, with those having primary incomplete education showing higher adjusted odds than those with no education (aOR: 1.37, 95% CI: 1.08–1.74, p = 0.010), as did those with secondary and above (aOR: 1.34, 95% CI: 1.04–1.74, p = 0.026). Primary complete education was not significantly associated in the adjusted model (aOR: 1.21, 95% CI: 0.96–1.52, p = 0.104). Marital status was significantly associated with formal healthcare utilisation. Compared to never married individuals (adjusted predicted probability: 14.5%), those who were married or cohabiting (aOR: 1.63, 95% CI: 1.32–2.02, p < 0.001), divorced or separated (aOR: 1.53, 95% CI: 1.16–2.02, p = 0.003), and widowed (aOR: 1.60, 95% CI: 1.16–2.22, p = 0.004) all had significantly higher adjusted odds, with adjusted predicted probabilities of 21.2%, 20.2%, and 20.9% respectively.

**Fig 1:**
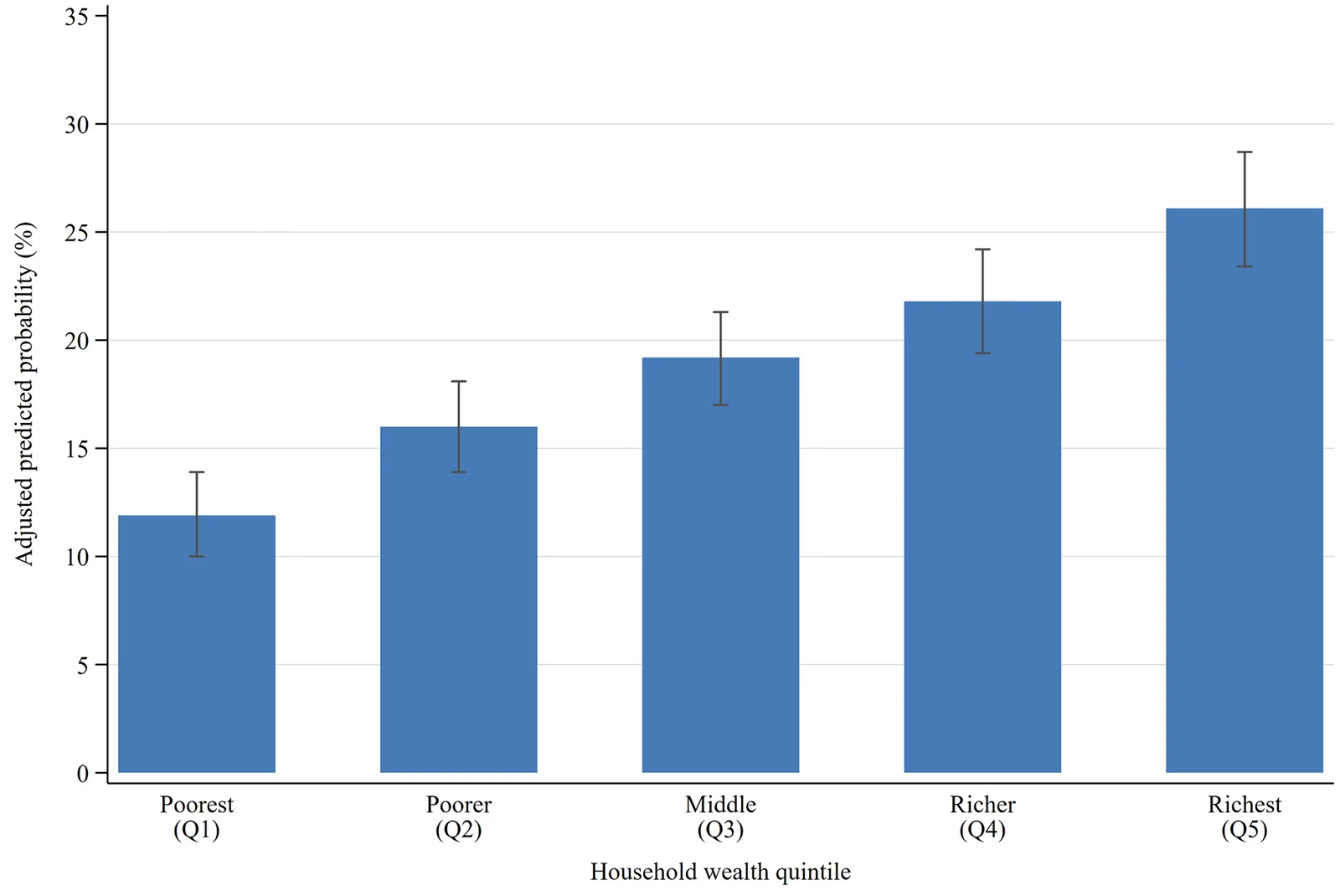
Survey-weighted adjusted predicted probabilities of formal healthcare utilisation by household wealth quintile (N = 11,891), Tanzania NPS 2020/2021.

Household wealth was the strongest and most consistent determinant of formal healthcare utilisation in both unadjusted and adjusted analyses. Each successive quintile had significantly higher adjusted odds than the poorest quintile, with a clear dose-response gradient: adjusted ORs rose from 1.42 (Q2) to 1.79 (Q3) to 2.12 (Q4) to 2.71 (Q5), all p ≤ 0.004. The adjusted predicted probability of formal healthcare utilisation rose from 11.9% in the poorest quintile to 26.1% in the richest, a difference of 14.2 percentage points that persisted after controlling for all other sociodemographic factors as shown on Fig 1.

Place of residence produced an important result. In the crude analysis, urban residents had 47% higher odds of formal healthcare utilisation than rural residents (crude OR: 1.47, 95% CI: 1.28–1.69, p < 0.001). After full adjustment, however, this association was entirely attenuated and became non-significant (aOR: 1.03, 95% CI: 0.85–1.24, p = 0.784), with adjusted predicted probabilities of 19.1% for rural and 19.4% for urban residents, a difference of only 0.3 percentage points. This pattern indicates that the apparent urban advantage in formal healthcare utilisation observed in bivariate analysis is largely explained by differences in the wealth, employment, and educational composition of urban and rural populations rather than by any independent effect of urban residence. Geographic zone was independently associated with formal healthcare utilisation after adjustment, with Southern Highlands being the only zone with significantly higher utilisation than the Lake zone reference (aOR: 2.22, 95% CI: 1.76–2.81, p < 0.001), corresponding to an adjusted predicted probability of 30.2% compared to 16.9% in the Lake zone. No other zone differed significantly from Lake zone in the adjusted model. Employment sector was not significantly associated with formal healthcare utilisation in the adjusted model (all p > 0.23). Proxy-identified mental health need remained non-significant in the adjusted model (aOR: 1.27, 95% CI: 0.53–3.04, p = 0.588). The adjusted predicted probabilities were 19.2% for those without proxy-identified mental health need and 22.9% for those with it (95% CI: 8.7–37.0%), a difference that was not statistically significant and had a very wide confidence interval reflecting the small observed subgroup of n = 54.

### Formal healthcare utilisation among individuals with proxy-identified mental health need

Of the 11,891 individuals in the regression analytic sample, 54 had proxy-identified mental health need with non-missing outcome data. This subgroup represents the observable component of a broader population with probable cognitive functional difficulty; a further 505 individuals had missing data on both the outcome and the proxy-identified mental health need item (Missing Not At Random group) and could not be included. All findings in this section are explicitly exploratory and are reported with their confidence intervals to reflect the high degree of uncertainty arising from the small sample size. Table 3 presents the weighted sociodemographic profile and formal healthcare utilisation patterns of this subgroup.

**Table 3:** Weighted sociodemographic profile and formal healthcare utilisation of individuals with proxy-identified mental health need (n = 54) compared to those without (n = 11,837), Tanzania NPS 2020/2021.

Characteristic	With proxy-identified mental health need (n = 54) Weighted % (95% CI)	Without proxy-identified mental health need (n = 11,837) Weighted % (95% CI)
**Formal healthcare utilisation**	22.0 (10.8–39.7)	19.2 (18.1–20.3)
**Mean age, years (95% CI)**	43.0 (35.8–50.3)	34.2 (33.9–34.6)
**Age group (years)**		
15–24	28.7 (16.4–45.4)	33.6 (32.6–34.6)
25–34	22.2 (10.6–40.6)	24.3 (23.2–25.5)
35–44	9.3 (3.7–21.7)	18.2 (17.2–19.2)
45–54	8.7 (3.1–22.6)	12.0 (11.2–12.7)
≥ 55	31.0 (18.6–47.0)	11.9 (11.1–12.8)
**Sex**		
Male	49.7 (33.6–65.9)	47.8 (46.9–48.6)
Female	50.3 (34.2–66.4)	52.2 (51.4–53.1)
**Place of residence**		
Rural	74.8 (58.7–86.1)	72.0 (69.9–74.0)
Urban	25.2 (13.9–41.3)	28.0 (26.0–30.1)
**Wealth quintile**		
Poorest (Q1)	23.6 (12.4–40.3)	19.8 (17.8–22.0)
Poorer (Q2)	12.9 (5.9–25.7)	20.3 (18.6–22.1)
Middle (Q3)	22.2 (12.2–37.0)	19.9 (18.3–21.6)
Richer (Q4)	23.5 (12.1–40.5)	19.9 (18.4–21.6)
Richest (Q5)	17.9 (7.3–37.3)	20.1 (18.2–22.0)
**Education level**		
No education	58.1 (41.3–73.2)	16.3 (14.9–17.9)
Primary incomplete	22.7 (11.3–40.6)	27.0 (25.9–28.1)
Primary complete	6.0 (2.3–14.8)	38.6 (37.1–40.2)
Secondary and above	13.1 (4.4–33.2)	18.1 (16.9–19.4)
**Marital status**		
Never married	55.1 (39.5–69.8)	31.2 (30.0–32.4)
Married/cohabiting	20.8 (10.2–37.8)	55.6 (54.1–57.1)
Divorced/separated	6.3 (1.8–20.2)	7.6 (6.9–8.3)
Widowed	17.9 (9.4–31.4)	5.7 (5.2–6.2)
**Employment sector**		
Formal wage	0.0 –	2.0 (1.6–2.5)
Informal wage	0.0 –	17.3 (16.2–18.5)
Self-employed non-farm	8.1 (3.1–19.8)	16.8 (15.7–18.0)
Agricultural	8.1 (2.4–24.0)	48.4 (46.5–50.4)
Not working/other	83.7 (68.6–92.4)	15.5 (14.4–16.6)
**Geographic zone**		
Lake	30.4 (18.0–46.6)	26.9 (25.2–28.7)
Western	5.7 (1.6–18.0)	9.8 (8.8–10.9)
Northern	15.8 (7.4–30.3)	13.7 (12.2–15.3)
Central	4.3 (1.1–15.9)	6.4 (5.5–7.5)
Eastern	25.9 (13.8–43.4)	19.2 (17.6–20.9)
Southern Highlands	15.8 (6.8–32.5)	15.8 (14.3–17.4)
Southern	1.9 (0.4–9.3)	5.2 (4.5–6.0)
Zanzibar	0.3 (0.1–1.4)	3.1 (2.8–3.4)

**Abbreviations:** CI, confidence interval; MH, mental health; NPS, National Panel Survey.

The subgroup had a notably older age profile than the full analytic sample. The mean weighted age was 43.0 years (95% CI: 35.8–50.3), approximately 8.7 years older than the full sample mean of 34.3 years. Those aged 55 and above accounted for 31.0% of the subgroup (95% CI: 18.6–47.0%), compared with 12.0% in the full analytic sample, while those aged 15–24 accounted for 28.7% (95% CI: 16.4–45.4%), compared with 33.6% in the full sample. The sex distribution was approximately equal (male 49.7%, female 50.3%), in contrast to the slight female majority in the full sample. The subgroup was predominantly rural (74.8%, 95% CI: 58.7–86.1%), with wealth broadly distributed across quintiles though with greater concentration in the poorest quintile (23.6%) and relatively lower representation in Q2 (12.9%). Levels of formal education were markedly lower in the subgroup than in the full analytic sample. An estimated 58.1% of individuals with proxy-identified mental health need had no formal education (95% CI: 41.3–73.2%), compared with 16.5% in the full sample, while secondary and above education was estimated at 13.1% (95% CI: 4.4–33.2%), compared with 18.1% in the full sample. Employment patterns also differed substantially: an estimated 83.7% were in the not working or other category (95% CI: 68.6–92.4%), compared with 15.8% in the full analytic sample, with no individuals in formal or informal wage employment.

Among the 54 individuals with proxy-identified mental health need and non-missing outcome data, the overall weighted formal healthcare utilisation was 22.0% (95% CI: 10.8–39.7%), with 9 individuals recorded as having visited a formal provider and 45 having not done so. The very wide confidence interval reflects the small sample size and limits interpretation. No statistically significant differences in formal healthcare utilisation were observed across any sociodemographic characteristic within this subgroup, which is expected given the small number of events.

To assess the sensitivity of this estimate to assumptions about the 505 individuals with missing data, extreme bounds analysis was conducted. Under the most conservative assumption that none of the 505 missing individuals would have visited a formal provider (lower bound, S1), weighted formal healthcare utilisation among all individuals with probable proxy-identified mental health need was 2.0% (95% CI: 0.9–4.4%). Under the most optimistic assumption that all 505 would have visited a formal provider (upper bound, S2), it was 92.7% (95% CI: 89.9–94.8%). The extreme width of this range, from 2.0% to 92.7% confirms that the observed estimate of 22.0% cannot be used to draw meaningful conclusions about the true level of formal healthcare utilisation among individuals with proxy-identified mental health need in Tanzania. Full sensitivity analysis results are presented in S2 Table.

## Discussion

This study provides a nationally representative population-level examination of formal healthcare utilisation among Tanzanians aged 15 years and above, drawing on the fifth wave of the National Panel Survey and an a priori Andersen Behavioural Model framework. Three principal findings stand out. First, approximately four in five Tanzanians had no contact with a formal health provider in the given four-week period. Second, formal healthcare utilisation in the stated period was driven primarily by structural enabling factors, household wealth, sex, age, marital status, and geographic zone rather than by proxy-identified mental health need. Third, severe difficulty remembering or concentrating was not independently associated with formal healthcare utilisation in either crude or adjusted analyses, a finding consistent with the broader literature on help-seeking behaviour in sub-Saharan Africa, but that must be interpreted with caution given the severe missing data limitations affecting the subgroup.

The wealth gradient in formal healthcare utilisation is the central finding of this study. The 14.2 percentage-point difference in adjusted predicted probabilities between the poorest and richest quintiles corresponding to adjusted odds 2.71 times higher in the richest quintile persisted after controlling for all covariates. This reflects the combined effect of direct costs including consultation fees, medicines, and diagnostics, indirect costs including transport and lost wages, and the chronic resource constraints that prevent poor households from prioritising illness care [4,31]. Demand-side financing mechanisms such as the Community Health Fund in Tanzania have not effectively reached the lowest wealth quintiles, and these findings add weight to calls for more universal social health protection schemes [32, 33]. The use of survey-weighted adjusted predicted probabilities translates this gradient into terms directly interpretable for policymakers and we encourage their routine reporting alongside odds ratios in similar analyses.

The female advantage in formal healthcare utilisation 22.4% versus 15.6% for men after full adjustment is consistent with prior evidence across sub-Saharan Africa and likely reflects women’s higher contact with formal health services through reproductive health programmes and greater willingness to seek care [3,9]. The converse finding, that men are substantially less likely to engage with formal health services, has implications for health system design and for the potential under-detection of health conditions including mental disorders among men in Tanzania.

The attenuation of the urban-rural difference after full adjustment is analytically important. Urban residents had 47% higher crude odds of formal healthcare utilisation, but after adjustment this was entirely attenuated and became non-significant (adjusted OR: 1.03, p = 0.784), with adjusted predicted probabilities of 19.1% rural versus 19.4% urban. Much of what appears as a geographic access effect in unadjusted data is better understood as a wealth and employment effect. Improving physical proximity of services alone may not close the utilisation gap if underlying socioeconomic barriers remain in place [3,9]. Southern Highlands zone showed the only significant independent zone effect (adjusted OR: 2.22, adjusted predicted probability: 30.2%), likely reflecting the density and quality of mission-based and government facilities in that region [34].

Regarding the null finding for proxy-identified mental health need, this study examined formal healthcare utilisation defined as any formal provider visit in the preceding four weeks prior to the survey interview regardless of reason. A person with severe difficulty remembering or concentrating who attended a clinic for malaria, injury, antenatal care, or hypertension would be counted as a utiliser. The null association (adjusted OR: 1.27, 95% CI: 0.53–3.04, p = 0.588) therefore does not speak to whether individuals with cognitive difficulty are more or less likely to seek care specifically for their cognitive symptoms; it speaks only to their overall formal provider contact rate [10]. This distinction is important for interpreting the finding. The null association is nevertheless consistent with evidence that cognitive and psychological symptoms in low-income African settings are frequently attributed to spiritual or social causes, managed through traditional and faith-based systems, or not recognised as problems warranting formal care [4,11]. These mechanisms are plausible but cannot be attributed with the available data.

The proxy-identified mental health need subgroup findings are entirely exploratory. The observable sample of 54 individuals is too small for reliable inference, and the extreme bounds analysis, formal healthcare utilisation ranging from 2.0% to 92.7% under different assumptions about the 505 individuals with missing data confirms that no conclusion about the subgroup’s true utilisation rate is possible.

This study has several limitations. The outcome captures any formal provider visit regardless of reason, precluding conclusions about mental health-specific care-seeking. The exposure is a single Washington Group item that serves as a proxy for cognitive functional impairment; it has not been validated as a clinical screening instrument in Tanzania and does not distinguish between depression, anxiety, neurodevelopmental conditions, dementia, or neurological impairment. Missing outcome data were concentrated in a Missing Not At Random pattern, and the direction of any resulting bias in subgroup estimates cannot be determined. The cross-sectional design precludes causal inference. The exclusion of the urban booster sample, while methodologically necessary for design-based inference, may limit generalisability to urban populations in the five affected regions.

## Conclusion

In this nationally representative study of 11,891 Tanzanians aged 15 years and above, formal healthcare utilisation defined as any formal provider visit in the preceding four weeks prior to the survey interview, regardless of reason was low overall at 19.2%, and was shaped predominantly by structural enabling factors. Household wealth was the strongest independent determinant, with a persistent 14.2 percentage-point gradient in adjusted predicted probabilities between the poorest and richest quintiles after full adjustment for all covariates. Female sex, older age, marital status beyond never married, and Southern-Highlands zone residence were also independently associated with higher formal healthcare utilisation. The apparent urban advantage in crude analysis was entirely explained by differences in the wealth and demographic composition of urban populations, confirming that socioeconomic rather than geographic factors are the primary drivers of formal healthcare engagement in Tanzania.

Severe difficulty remembering or concentrating used here as a proxy indicator of mental health need, was not independently associated with formal healthcare utilisation in the period studied, a null finding that must be interpreted carefully given that this study measured general formal healthcare contact regardless of the reason for the visit, and given the severe missing data limitations affecting the subgroup. No inference about mental health-specific care-seeking or the true level of formal healthcare utilisation among individuals with cognitive functional difficulty is possible from these data alone.

Reducing financial barriers for the poorest households and strengthening equitable geographic distribution of formal health services are the most directly supported policy priorities arising from this study’s findings. The relationship between cognitive functional difficulty and formal healthcare utilisation in Tanzania requires further investigation using validated mental health screening instruments, longitudinal designs, and larger population samples before policy recommendations specific to this group can be made with confidence.

## Supporting information

S1 Table Extreme bounds sensitivity analysis: formal healthcare utilisation among individuals with proxy-identified mental health need under three assumptions about missing data, Tanzania NPS 2020/2021. (DOCX)

S2 Table STROBE Checklist for cross-sectional studies. (DOCX)
