# Supplementary material for: Patterns and determinants of formal healthcare utilisation among individuals with and without proxy-identified mental health need in Tanzania: A cross-sectional analysis of the National Panel Survey
Source: PLOS Ment Health. 2026 Sep 17;3(9):e0000644. doi: 10.1371/journal.pmen.0000644 (PMC13585274; doi:10.1371/journal.pmen.0000644)
Supplement: Supplementary file 1 [file pmen.0000644.s001.docx]

**S1 Table: Extreme bounds sensitivity analysis: formal healthcare utilisation among individuals with proxy-identified mental health need under three assumptions about missing data, Tanzania NPS 2020/2021.**

| **Scenario** | **Assumption about 505 individuals with missing data** | **n included** | **Formal healthcare utilisation %**  **(95% CI)** | **Interpretation** |
| --- | --- | --- | --- | --- |
| S0 Observed | Only the 54 individuals with proxy-identified mental health need and non-missing outcome are included. No assumption is made about the 505. | 54 | 22.0% (10.8–39.7%) | The observable estimate under the assumption that only those with non-missing data are representative. This estimate is highly uncertain. |
| S1 Lower bound | All 505 individuals with missing data are assumed to have proxy-identified mental health need AND to have not visited a formal provider (most pessimistic assumption). | 559 | 2.0% (0.9–4.4%) | The minimum plausible estimate if all missing individuals had cognitive difficulty and did not utilise formal healthcare. |
| S2 Upper bound | All 505 individuals with missing data are assumed to have proxy-identified mental health need AND to have visited a formal provider (most optimistic assumption). | 559 | 92.7% (89.9–94.8%) | The maximum plausible estimate if all missing individuals had cognitive difficulty and did utilise formal healthcare. |
