# Supplementary material for: Patterns and determinants of formal healthcare utilisation among individuals with and without proxy-identified mental health need in Tanzania: A cross-sectional analysis of the National Panel Survey
Source: PLOS Ment Health. 2026 Sep 17;3(9):e0000644. doi: 10.1371/journal.pmen.0000644 (PMC13585274; doi:10.1371/journal.pmen.0000644)
Supplement: Supplementary file 2 [file pmen.0000644.s002.docx]

S2 Table: Completed STROBE checklist for cross-sectional studies

This checklist was elaborated using formal items recommended for cross-sectional studies from the STROBE statement (<https://www.strobe-statement.org>).

|  | **Item No** | **Recommendation** | **Respected?** | **Comments and quotes** |
| --- | --- | --- | --- | --- |
| **Title and abstract** | 1 | (*a*) Indicate the study’s design with a commonly used term in the title or the abstract | Yes | The study design is clearly stated in the title. “Patterns and determinants of formal healthcare utilisation among individuals with and without proxy-identified mental health need in Tanzania: A cross-sectional analysis of the National Panel Survey.” Page 1 |
|  |  | (*b*) Provide in the abstract an informative and balanced summary of what was done and what was found | Yes | The abstract provides an unstructured single-paragraph summary covering background, data source, analytic sample, methods, key findings including the wealth gradient and null finding for proxy-identified mental health need, sensitivity analysis range, and conclusions. |
| **Introduction** | | | | |
| Background/rationale | 2 | Explain the scientific background and rationale for the investigation being reported | Yes | The introduction provides scientific background on the global and Tanzanian burden of mental health disorders, determinants of formal healthcare utilisation, proxy justification, and gaps in existing evidence as rationale. |
| Objectives | 3 | State-specific objectives, including any prespecified hypotheses | Yes | Three specific objectives are explicitly stated at the end of the Introduction: first, to describe the prevalence and sociodemographic patterning of formal healthcare utilisation in the full analytic sample; second, to identify independent determinants of formal healthcare utilisation using multivariable logistic regression within the Andersen Behavioural Model framework; and third, to describe the profile and formal healthcare utilisation of the subgroup with proxy-identified mental health need, with explicit acknowledgement of the data limitations affecting subgroup inference. |
| **Methods** | | | | |
| Study design | 4 | Present key elements of study design early in the paper | Yes | Study design is presented in the “Study design and data source” subsection of the Materials and methods section. “This is a secondary cross-sectional analysis using data from the fifth wave of the Tanzania National Panel Survey (NPS Wave 5, 2020/21), a nationally representative household survey conducted between 2020 and 2021 by the National Bureau of Statistics of Tanzania in partnership with the World Bank Living Standards Measurement Study programme.” |
| Setting | 5 | Describe the setting, locations, and relevant dates, including periods of recruitment, exposure, follow-up, and data collection | Yes | The setting, country, survey period, and sampling framework are described in the Materials and methods section under the Study design and data source heading. The NPS Wave 5 employed a stratified two-stage cluster sampling design, with households selected through probability proportional to size sampling from enumeration areas defined in the 2012 national census, covering 51 strata across mainland Tanzania and Zanzibar. |
| Participants | 6 | (*a*) Give the eligibility criteria, and the sources and methods of selection of participants | Yes | Eligibility criteria, sample derivation, and all exclusion steps are described in the Study population subsection. All individuals aged 15 years and above were eligible. The urban booster sample (n = 1,296) was excluded due to missing primary sampling unit and stratum identifiers. A further 505 individuals with missing outcome data were excluded, yielding a final regression analytic sample of 11,891 individuals representing a weighted population of 31,050,573. |
| Variables | 7 | Clearly define all outcomes, exposures, predictors, potential confounders, and effect modifiers. Give diagnostic criteria, if applicable | Yes | The primary outcome (formal healthcare utilisation), primary exposure (proxy-identified mental health need using the Washington Group Short Set severe threshold), and all nine covariates are defined in the Outcome variable, Exposure variable, and Covariates subsections of the Methods. Three excluded variables (insurance, distance to facility, recent illness) are explicitly justified. |
| Data sources/ measurement | 8* | For each variable of interest, give sources of data and details of methods of assessment (measurement). Describe comparability of assessment methods if there is more than one group | Yes | All variables are drawn from the Tanzania NPS Wave 5 household survey. Source and measurement methods are described in the Study design and data source, Outcome variable, Exposure variable, and Covariates subsections. The outcome is a self-reported measure, with responses provided by the individual or, where the individual was unavailable, by a knowledgeable household member acting as a proxy respondent, as stated explicitly in the Outcome variable subsection. All variables were measured identically for every individual through the same questionnaire administered at the same time. |
| Bias | 9 | Describe any efforts to address potential sources of bias | Yes | Potential sources of bias are addressed through: survey-weighted analysis accounting for the complex sampling design; a priori model specification using the Andersen Behavioural Model; transparent documentation of the Missing Not At Random pattern; and extreme bounds sensitivity analysis to bound the range of plausible subgroup estimates. |
| Study size | 10 | Explain how the study size was arrived at | Yes | This is a secondary analysis; no a priori sample size calculation was performed. Study size is determined by the Tanzania NPS Wave 5 sampling frame. The derivation from the full dataset to the final analytic sample is documented step by step in the Study population subsection. |
| Quantitative variables | 11 | Explain how quantitative variables were handled in the analyses. If applicable, describe which groupings were chosen and why | Yes | The outcome (formal healthcare utilisation) was treated as a binary variable. The proxy-identified mental health need exposure was dichotomised at the severe threshold (codes 4-5 on the 1-5 scale) consistent with Washington Group recommendations. Age was grouped into five categories. Household wealth was categorised into quintiles from survey-weighted real consumption expenditure per adult equivalent. All groupings are described with rationale in the Methods. |
| Statistical methods | 12 | (*a*) Describe all statistical methods, including those used to control for confounding | Yes | All statistical methods are described in the Data analysis subsection, including survey-weighted descriptive statistics, Rao-Scott design-based F-tests, and survey-weighted multivariable logistic regression with all nine predictors included simultaneously as the adjusted model. The Andersen Behavioural Model provided the a priori theoretical basis for covariate selection. |
|  |  | (*b*) Describe any methods used to examine subgroups and interactions | Yes | Subgroup analysis of the proxy-identified mental health need group was conducted using weighted descriptive methods. Multivariable regression was not pursued in the subgroup due to the very small number (n = 54) with non-missing outcome data. All subgroup findings are explicitly framed as exploratory. |
|  |  | (*c*) Explain how missing data were addressed | Yes | Missing data handling is described in the Missing data subsection of the Methods. Individuals with missing outcome values were excluded. The pattern of missingness (perfectly concentrated in those with missing proxy-identified mental health need) is characterised as Missing Not At Random. |
|  |  | (*d*) If applicable, describe analytical methods taking account of sampling strategy | Yes | Survey design is fully accounted for using Stata’s svyset command incorporating household sampling weights, PSU clustering, and stratification throughout all analyses. The singleunit(centered) option was applied to handle strata with single PSUs. This is described in the Data analysis subsection.. |
|  |  | (*e*) Describe any sensitivity analyses | Yes | Extreme bounds sensitivity analysis was conducted to assess the implications of Missing Not At Random data for subgroup estimates. Three scenarios were constructed: the observed estimate using 54 individuals with non-missing outcome (S0: 22.0%, 95% CI: 10.8–39.7%); a lower bound assuming all 505 missing individuals did not visit a formal provider (S1: 2.0%, 95% CI: 0.9–4.4%); and an upper bound assuming all 505 did visit (S2: 92.7%, 95% CI: 89.9–94.8%). Results are presented in S1 Table. |
| **Results** | | | | |
| Participants | 13* | (*a*) Report numbers of individuals at each stage of study—eg numbers potentially eligible, examined for eligibility, confirmed eligible, included in the study, completing follow-up, and analysed | Yes | Numbers at each stage are reported in the Results section: full dataset (23,592), aged under 15 excluded (-9,900), aged 15+ (13,692), urban booster sample excluded (-1,296), main analytic sample (12,396), missing outcome excluded (-505), regression analytic sample (11,891), proxy-identified mental health need subgroup with non-missing outcome (n = 54). |
|  |  | (*b*) Give reasons for non-participation at each stage | Yes | Reasons for all exclusions are described in the Study population and Missing data subsections of the Methods and restated in the Results. Booster exclusion: missing primary sampling unit and stratum identifiers. Outcome missing: Missing Not At Random pattern, possible data collection difficulty. |
|  |  | (*c*) Consider use of a flow diagram | N/A | A flow diagram was not included. Sample derivation is clearly described in text in the Study population subsection and in the Results with all numbers transparently reported. Given the straightforward three-step exclusion process, a flow diagram was not considered necessary. |
| Descriptive data | 14* | (*a*) Give characteristics of study participants (eg demographic, clinical, social) and information on exposures and potential confounders | Yes | Weighted sociodemographic characteristics of all participants are presented in Table 1, including distributions by age group, sex, residence, wealth quintile, education level, marital status, employment sector, and geographic zone, alongside formal healthcare utilisation prevalence for each group. |
|  |  | (*b*) Indicate number of participants with missing data for each variable of interest | Yes | Missing data are reported in the Study population and Missing data subsections of the Methods and in the Results. Only the outcome variable had missing values. The proxy-identified mental health need variable had the same 505 missing values as the outcome. All other variables had complete data in the analytic sample. |
| Outcome data | 15* | Report numbers of outcome events or summary measures | Yes | The weighted prevalence of formal healthcare utilisation is reported for the full analytic sample (19.2%, 95% CI: 18.1–20.3%) and for all sociodemographic subgroups in Table 1, and for individuals with proxy-identified mental health need in Table 3. The outcome is defined as any formal provider visit in the four weeks prior to interview, regardless of reason. |
| Main results | 16 | (*a*) Give unadjusted estimates and, if applicable, confounder-adjusted estimates and their precision (eg, 95% confidence interval). Make clear which confounders were adjusted for and why they were included | Yes | Crude ORs, adjusted ORs, and 95% CIs are presented side by side in Table 2 for all nine predictors. The adjusted model included: proxy-identified mental health need, sex, age group, education level, marital status, wealth quintile, place of residence, geographic zone, and employment sector, all specified a priori within the Andersen Behavioural Model framework. |
|  |  | (*b*) Report category boundaries when continuous variables were categorized | Yes | Category boundaries for age groups (15-24, 25-34, 35-44, 45-54, 55+) and wealth quintiles (Q1 poorest to Q5 richest) are reported in Table 1, Table 2, and Table 3 with clear labels. |
|  |  | (*c*) If relevant, consider translating estimates of relative risk into absolute risk for a meaningful time period | Yes | Survey-weighted adjusted predicted probabilities are reported in Table 2 for all nine modelled predictors including place of residence, translating odds ratios into absolute probabilities of formal healthcare utilisation for each predictor category. These are also discussed throughout the Results and Discussion sections. |
| Other analyses | 17 | Report other analyses done—eg analyses of subgroups and interactions, and sensitivity analyses | Yes | A pre-planned weighted descriptive subgroup analysis of individuals with proxy-identified mental health need (n = 54) is reported in the Results and Table 3. Extreme bounds sensitivity analysis is reported in S1 Table with S0 (22.0%), S1 (2.0%), and S2 (92.7%) estimates. |
| **Discussion** | | | | |
| Key results | 18 | Summarise key results with reference to study objectives | Yes | The three principal findings are summarised at the opening of the Discussion with explicit reference to the three study objectives: overall utilisation level, structural determinants, and the null finding for proxy-identified mental health need. |
| Limitations | 19 | Discuss limitations of the study, taking into account sources of potential bias or imprecision. Discuss both direction and magnitude of any potential bias | Yes | Limitations are discussed in the final paragraphs of the Discussion section, covering: non-specific outcome (any formal visit regardless of reason); unvalidated single-item proxy; cross-sectional design precluding causal inference; MNAR missingness; small proxy MH need subgroup; and exclusion of the urban booster sample. |
| Interpretation | 20 | Give a cautious overall interpretation of results considering objectives, limitations, multiplicity of analyses, results from similar studies, and other relevant evidence | Yes | Cautious interpretation is maintained throughout the Discussion. The null finding for proxy-identified mental health need is interpreted carefully with reference to the wide confidence interval, the small sample size, and the fact that the outcome measures general formal provider contact regardless of reason rather than mental health-specific care-utilisation. |
| Generalisability | 21 | Discuss the generalisability (external validity) of the study results | Yes | The nationally representative NPS Wave 5 design supports generalisability to the Tanzanian population aged 15 and above. Caution for subgroup estimates is explicitly noted. The exclusion of the urban booster sample from five mainland regions is acknowledged as a potential limitation for urban generalisability. |
| **Other information** | | | | |
| Funding | 22 | Give the source of funding and the role of the funders for the present study and, if applicable, for the original study on which the present article is based | Yes | The authors received no specific funding for this work. This statement appears in the Funding section of the published manuscript. |

*Give information separately for exposed and unexposed groups.

**Note:** An Explanation and Elaboration article discusses each checklist item and gives methodological background and published examples of transparent reporting. The STROBE checklist is best used in conjunction with this article (freely available on the Web sites of PLoS Medicine at http://www.plosmedicine.org/, Annals of Internal Medicine at http://www.annals.org/, and Epidemiology at http://www.epidem.com/). Information on the STROBE Initiative is available at www.strobe-statement.org.
